# IgA vasculitis induced by carboplatin + nab-paclitaxel + pembrolizumab in a patient with advanced lung squamous cell carcinoma: a case report

**DOI:** 10.3389/fimmu.2024.1370972

**Published:** 2024-08-14

**Authors:** Yuto Terashima, Masaru Matsumoto, Saeko Ozaki, Michiko Nakagawa, Shun Nakagome, Yasuhiro Terasaki, Hiroki Iida, Ryotaro Mitsugi, Eri Kuramochi, Naoko Okada, Tomoyasu Inoue, Satoru Matsuki, Shingo Kitagawa, Aya Fukuizumi, Naomi Onda, Susumu Takeuchi, Akihiko Miyanaga, Kazuo Kasahara, Masahiro Seike

**Affiliations:** ^1^ Department of Pulmonary Medicine and Oncology, Graduate School of Medicine, Nippon Medical School, Tokyo, Japan; ^2^ Department of Dermatology, Graduate School of Medicine, Nippon Medical School, Tokyo, Japan; ^3^ Department of Gastroenterology, Graduate School of Medicine, Nippon Medical School, Tokyo, Japan; ^4^ Department of Analytic Human Pathology, Graduate School of Medicine, Nippon Medical School, Tokyo, Japan

**Keywords:** IgA vasculitis, immune-related adverse event, immune checkpoint inhibitor, pembrolizumab, non-small-cell lung cancer

## Abstract

A 73-year-old man with lung squamous cell carcinoma was administered carboplatin + nab-paclitaxel + pembrolizumab for four cycles. Subsequently, he presented with multiple purpuras on his extremities, joint swelling on his fingers, abdominal pain, and diarrhea, accompanied by acute kidney injury (AKI), increased proteinuria, hematuria, and elevated C-reactive protein levels. Skin biopsy showed leukocytoclastic vasculitis as well as IgA and C3 deposition in the vessel walls. Based on these findings, the patient was diagnosed with IgA vasculitis as an immune-related adverse event (irAE) induced by carboplatin + nab-paclitaxel + pembrolizumab. After discontinuation of pembrolizumab and glucocorticoids, the symptoms immediately resolved. Regular monitoring of skin, blood tests, and urinalysis are necessary, and the possibility of irAE IgA vasculitis should be considered in cases of purpura and AKI during treatment with immune checkpoint inhibitors.

## Introduction

Standard therapeutic options for lung cancer have recently expanded with the development of immune checkpoint inhibitors (ICIs) (1). ICIs can enhance T lymphocytes activity and promote anti-tumor responses by blocking immune checkpoints (2). However, due to these mechanisms, ICIs can cause various side effects, which are known as immune-related adverse events (irAEs). IrAEs can develop in organs throughout the body, such as dermatitis, thyroid dysfunction, colitis, nephritis and pneumonitis. On the other hand, irAE vasculitis is rare (3). We herein report a case of irAE IgA vasculitis induced by carboplatin + nab-paclitaxel + pembrolizumab, an ICI.

## Case report

**Figure 1:**
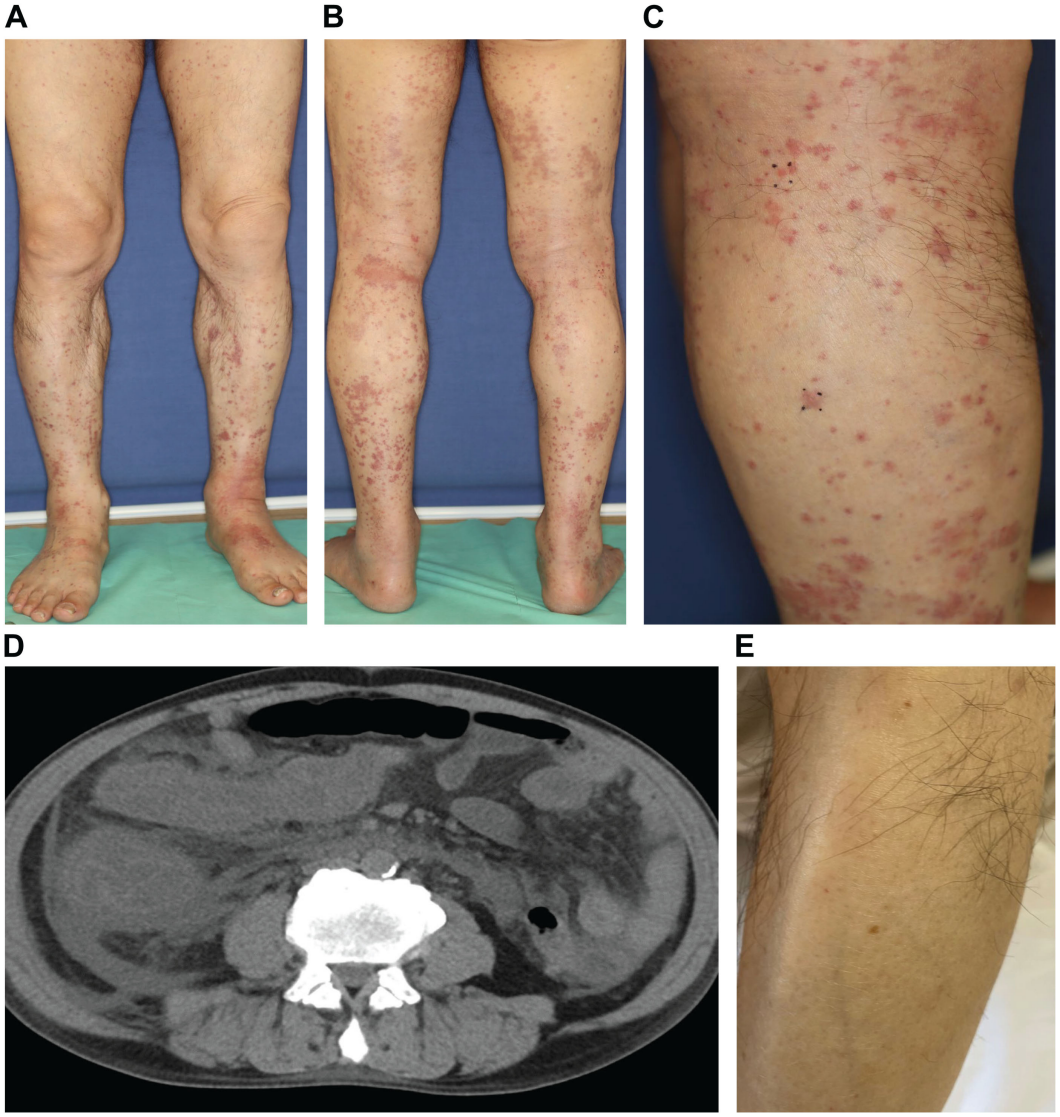
**(A–C)** The patient developed multiple purpuras on his extremities. **(D)** CT revealed intestinal edema and ascites. **(E)** The purpura improved immediately after glucocorticoid treatment.

**Figure 2:**
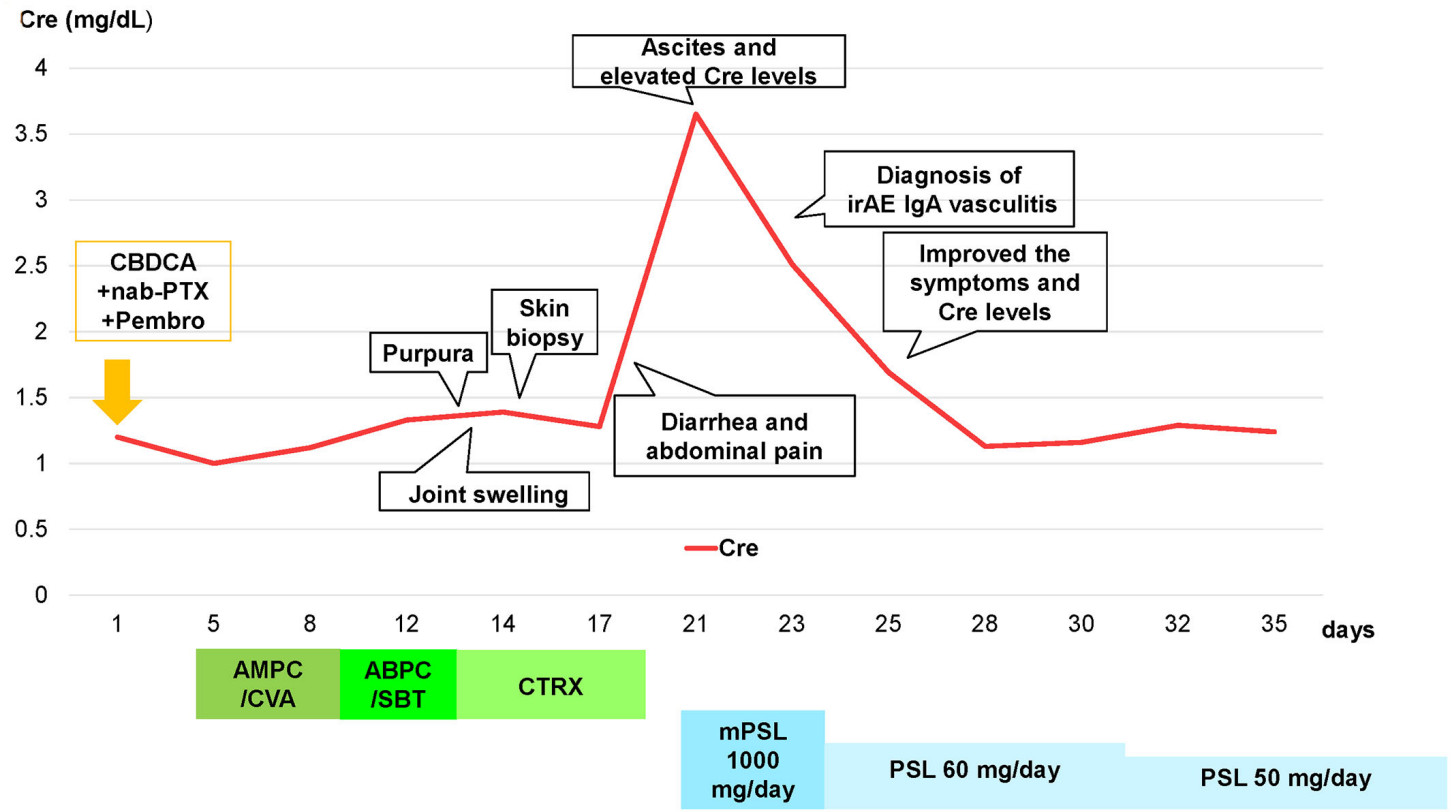
Clinical course of the patient and transition of creatinine levels after four cycles of carboplatin + nab-paclitaxel + pembrolizumab treatment, and diagnosed with irAE IgA vasculitis. Abbreviations: Cre, creatinine; CBDCA, carboplatin; nab-PTX, nab-paclitaxel; Pembro, pembrolizumab; AMPC/CVA, amoxicillin–clavulanate; ABPC/SBT, ampicillin–sulbactam; CTRX, ceftriaxone; mPSL, methylprednisolone; PSL, prednisolone.

**Figure 3:**
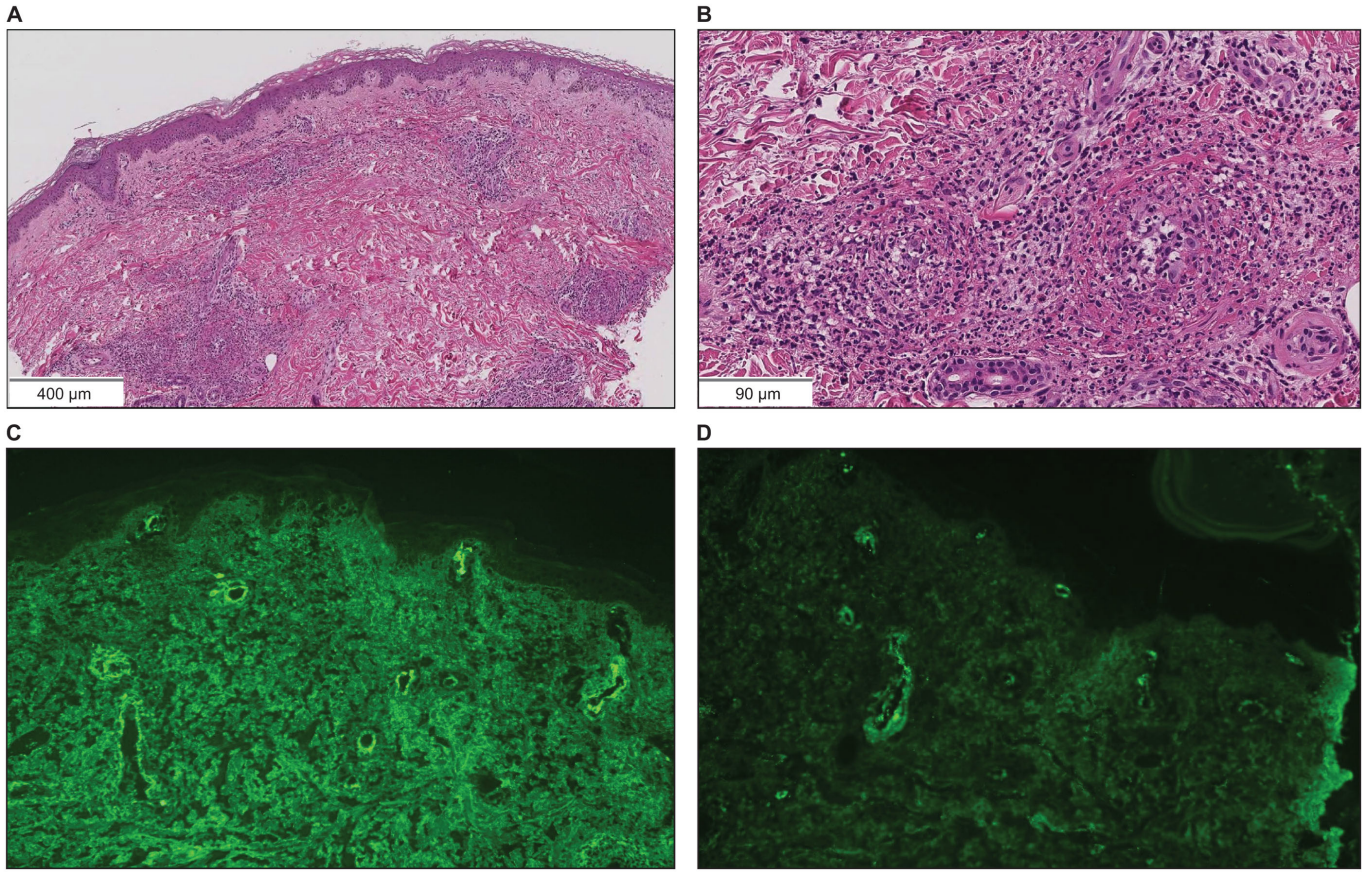
Skin biopsy findings on the lower leg. **(A, B)** Hematoxylin and eosin staining showed neutrophil-dominated inflammatory cell infiltration from the small vessel walls into the surrounding tissues (scale bar [A] = 400 µm, [B] = 90 µm). **(C)** Immunofluorescence staining revealed granular IgA deposition in the vessel walls (×200). **(D)** Immunofluorescence staining revealed granular C3 deposition in the vessel walls (×200).

A 73-year-old Japanese man presented with cough and dyspnea. Computed tomography (CT) showed a tumor in the left lower lobe, with lymph node enlargement at the left hilum and metastasis to the sacrum and left adrenal gland. We performed a transbronchial lung biopsy with bronchoscopy. Based on these findings and the result of biopsy, he was diagnosed with lung squamous cell carcinoma, cT4N2M1c stage IVB. The Oncomine™ Dx Target Test revealed no mutation of cancer-relevant genes, and PD-L1 expression was 10%–24% in the 22C3 assay. Before chemotherapy, the patient developed obstructive pneumonia and had an Eastern Cooperative Oncology Group Performance Status (ECOG-PS) score of 2. After antibacterial therapy for obstructive pneumonia, carboplatin and nab-paclitaxel were administered as first-line therapy. In the second treatment course, pembrolizumab was added as the patient’s ECOG-PS score improved. Five days after four cycles of treatment, the patient presented with fever and elevated C-reactive protein (CRP) levels. This led to the suspicion of recurrent obstructive pneumonia; thus, oral antibacterial therapy was initiated. Due to limited improvements in symptoms, the patient was admitted to our hospital. However, he developed multiple purpuras on his extremities and joint swelling on his fingers (**Figures 1A–C**). Purpura was observed not only on the lower legs but also on the thighs and abdomen. Therefore, we suspected irAEs and performed skin biopsy on the lower leg. Furthermore, additional symptoms of diarrhea and abdominal pain occurred. Laboratory analysis showed elevated white blood cells (11300/µL), CRP (17.66 mg/dL), blood urea nitrogen (30.3 mg/dL), and creatinine (3.35 mg/dL) levels as well as proteinuria and hematuria (**Figure 2**). In addition, serum IgA levels increased to 396 mg/dL. CT revealed intestinal edema and ascites (**Figure 1D**). A skin biopsy specimen exhibited neutrophil-dominated inflammatory cell infiltration from the small vessel walls into the surrounding tissues, suggestive of leukocytoclastic vasculitis (**Figures 3A, B**). Immunofluorescence staining showed granular IgA and C3 deposition in the vessel walls (**Figures 3C, D**). Therefore, the patient was diagnosed with irAE IgA vasculitis. We considered that symptoms such as purpura, joint swelling, acute kidney injury, and ascites were due to irAE IgA vasculitis. He was treated with intravenous methylprednisolone (1000 mg/day) followed by prednisolone (1 mg/kg/day) for 3 days. After the treatment initiation, the symptoms immediately resolved and creatinine levels improved (**Figures 1E**, **2**). Subsequently, we tapered prednisolone from 60 mg/day to 30 mg/day by reducing the dose by 10 mg/day each week. After that, we continued to taper the dose by 5 mg/day every two weeks. Currently, the patient is maintaining a dose of 5 mg/day of prednisolone, and there has been no exacerbation of symptoms. Moreover, for lung cancer, we are currently on a watchful waiting approach without chemoimmunotherapy, including pembrolizumab, and the disease has been stable for four months.

## Discussion

ICIs, including pembrolizumab, enhances T cell activity against tumors and leads to improved clinical outcomes in various cancers by blocking inhibitory pathways like PD-1/PD-L1. The restoration of immune activity can lead to irAEs, such as colitis, hepatitis, endocrinopathies, pneumonitis, and vasculitis (2). In previous reports, most cases of irAE vasculitis were large vessel vasculitis (3). Thus, small vessel vasculitis, such as IgA vasculitis, is rare. IrAE IgA vasculitis was reported in patients with advanced melanoma as well as hepatocellular, renal cell, and squamous cell lung carcinomas, as in our study (**Table 1**) (4–9). These cases were induced by nivolumab, ipilimumab and durvalumab. To the best of our knowledge, this is the first report of irAE IgA vasculitis induced by chemoimmunotherapy, including pembrolizumab.

**Table 1:** Previous cases of irAE IgA vasculitis and the present case.

No.	Author	Sex	Age	Cancer	Regimen	Time to onset	Affected organ	Treatment	Outcome	Readministration
1	Belkaid (4)	M	70	Melanoma	Nivolumab + Ipilimumab	7 months	Skin, gastrointestinal, joint, and renal	Discontinuation of ICI and GC	Remission	–
2	Mamlouk (5)	F	50s	Melanoma	Nivolumab + Ipilimumab	5 weeks	Skin, renal	Discontinuation of ICI, GC, rituximab, plasmapheresis, and hemodialysis	Chronic renal failure	–
3	Asemota (6)	M	60	HCC	Nivolumab	9 months	Skin, renal	Discontinuation of ICI and GC	Death (after 18 days)	–
4	Nagaoka (7)	F	50	RCC	Nivolumab	21 months	Skin, gastrointestinal, joint, and renal	Discontinuation of ICI and GC	Remission	+ (late line)
5	Casafont-Sole (8)	M	64	SCC	Durvalumab	6 months	Skin, renal	Discontinuation of ICI and GC	Remission	–
6	Kawataki (9)	M	78	SCC	Durvalumab	18 months	Skin, gastrointestinal, and renal	Discontinuation of ICI and GC	Remission	–
7	Present case	M	73	SCC	CBDCA + nab-PTX + Pembrolizumab	3 months	Skin, gastrointestinal, joint, and renal	Discontinuation of ICI and GC	Remission	–

F, female; M, male; HCC, hepatocellular carcinoma; RCC, renal cell carcinoma; SCC, squamous cell carcinoma; CBDCA, carboplatin; nab-PTX, nab-paclitaxel; ICI, immune checkpoint inhibitor; GC, glucocorticoid.

IgA vasculitis commonly occurs in children and is triggered by infections, tumors, drugs, and food (10, 11). In the present case, infections such as group A *streptococcus* or parvovirus, which are the frequent causes, were ruled out as antistreptolysin O antibody and parvovirus B19 were negative. Furthermore, the test results were negative for urinary tract, fungal, and other viral infections as well as tuberculosis. There was also no evidence of cancer progression. Obstructive pneumonia and administered antibiotics were suspected as causes. The patient had been previously treated with the same antibiotics (amoxicillin/clavulate and ampicillin/sulbactam), and he had never developed irAE IgA vasculitis. Moreover, a previous report suggested that the alteration of the PD-L1/PD-1 axis might be associated with the regulation of T-cell activation in the skin and peripheral blood of patients with vasculitis (12). Thus, although there is a possibility that the symptoms of irAE IgA vasculitis became apparent due to the concurrent administration of chemoimmunotherapy and antibiotics, we diagnosed the patient irAE IgA vasculitis induced by carboplatin + nab-paclitaxel + pembrolizumab.

In general, irAE has been managed with ICI discontinuation and systemic corticosteroid administration. If improvement is limited, immunosuppressive agents may be administered as the next treatment (13, 14). In previous reports, four out of six cases achieved remission with only discontinuation of ICIs and systemic corticosteroids (**Table 1**). In half of the reports, the treatment started with intravenous administration of methylprednisolone (500-1000 mg/day), followed by tapering to prednisolone (0.5-1 mg/kg/day). Our patient also rapidly improved with these treatments. Therefore, immunosuppressive agents were not administered. Further studies are warranted to establish the treatment strategy for irAE IgA vasculitis, which is relatively rare.

The clinical benefit of ICI readministration in patients with irAE IgA vasculitis remains unclear. In general, it is often not recommended in cases of grade 3 or higher irAEs (15). In previous reports, ICIs were not readministered in five of six cases after treatment for irAE IgA vasculitis (**Table 1**). In one case of ICI readministration, the treatment was conducted under prednisolone therapy, and no recurrence of irAEs was observed. Our patient developed grade 3 irAE IgA vasculitis and we determined that readministration of ICIs carries a high risk, even under steroid therapy. Furthermore, CT revealed no evidence of cancer progression. Thus, we did not readminister pembrolizumab. If the lung cancer progresses in the future, we plan to administer non-ICI drugs such as docetaxel.

In summary, we present a case of irAE IgA vasculitis induced by carboplatin + nab-paclitaxel + pembrolizumab. Regular monitoring of skin, blood tests, and urinalysis are necessary, and the possibility of irAE IgA vasculitis should be considered in cases of purpura and acute kidney injury during ICI treatment.

## Data Availability

The original contributions presented in the study are included in the article/supplementary material. Further inquiries can be directed to the corresponding author.

## References

[1] Paz-Ares L Luft A Vicente D Tafreshi A Gümüş M Mazières J. Pembrolizumab plus chemotherapy for squamous non–small-cell lung cancer. New Engl J Med. (2018) 379:2040–51. doi: 10.1056/NEJMoa1810865 30280635

[2] Abdel-Wahab N Shah M Suarez-Almazor ME. Adverse events associated with immune checkpoint blockade in patients with cancer: A systematic review of case reports. PloS One. (2016) 11:e0160221. doi: 10.1371/journal.pone.0160221 27472273 PMC4966895

[3] Daxini A Cronin K Sreih AG. Vasculitis associated with immune checkpoint inhibitors—a systematic review. Clin Rheumatol Springer London;. (2018) 37:2579–84. doi: 10.1007/s10067-018-4177-0 29923081

[4] Belkaid S Berger M Nouvier M Picard C Dalle S. A case of Schönlein–Henoch purpura induced by immune checkpoint inhibitor in a patient with metastatic melanoma. Eur J Cancer. (2020) 139:169–72. doi: 10.1016/j.ejca.2020.08.005 32992155

[5] Mamlouk O Lin JS Abdelrahim M Tchakarov AS Glass WF Selamet U. Checkpoint inhibitor-related renal vasculitis and use of rituximab. J Immunother Cancer. (2020) 8:e000750. doi: 10.1136/jitc-2020-000750 32718987 PMC7380836

[6] Asemota U Gulati A Kumar K Janga K. Nivolumab-induced crescentic immunoglobulin A nephropathy with henoch-schonlein purpura features in a patient diagnosed with hepatocellular carcinoma. Cureus. (2021) 13:e19110. doi: 10.7759/cureus.19110 34868760 PMC8627579

[7] Nagaoka-Takatori A Ishii M Hayama K Obinata D Yamaguchi K Takahashi S. A case of igA vasculitis during nivolumab therapy for renal cell carcinoma. Clin Cosmet Investig Dermatol. (2021) 14:1885–8. doi: 10.2147/CCID.S343876 PMC8713720 34992403

[8] Casafont-Solé I Martínez-Morillo M Camins-Fàbregas J Brandy-García A Quer A Moran T. IgA vasculitis and polymyalgia rheumatica induced by durvalumab. Trans Lung Cancer Res. (2020) 9:421–3. doi: 10.21037/tlcr PMC7225140 32420087

[9] Kawataki M Watanabe K Yokoyama T Ishida T. IgA vasculitis as an immune-related adverse event of durvalumab: A case report. Respir Investig. (2023) 61:205–9. doi: 10.1016/j.resinv.2023.01.005 36773508

[10] Song Y Huang X Yu G Qiao J Cheng J Wu J. Pathogenesis of igA vasculitis: an up-to-date review. Front Immunol. (2021) 12. doi: 10.3389/fimmu.2021.771619 PMC8630619 34858429

[11] Xu L Li Y Wu X. IgA vasculitis update: Epidemiology, pathogenesis, and biomarkers. Front Immunol. (2022) 13. doi: 10.3389/fimmu.2022.921864 PMC9574357 36263029

[12] Miyabe C Dong Y Ikeda T Takahashi K Miyabe Y Kawakami T. Immune checkpoint molecule expression is altered in the skin and peripheral blood in vasculitis. Sci Rep. (2021) 11:20019. doi: 10.1038/s41598-021-99558-5 34625602 PMC8501116

[13] Leipe J Mariette X. Management of rheumatic complications of ICI therapy: A rheumatology viewpoint. Rheumatol (United Kingdom). (2019) 58:vii49–58. doi: 10.1093/rheumatology/kez360 PMC6900914 31816078

[14] Baraibar I Melero I Ponz-Sarvise M Castanon E. Safety and tolerability of immune checkpoint inhibitors (PD-1 and PD-L1) in cancer. Drug Saf. (2019) 42:281–94. doi: 10.1007/s40264-018-0774-8 30649742

[15] Haanen J Ernstoff M Wang Y Menzies A Puzanov I Grivas P. Rechallenge patients with immune checkpoint inhibitors following severe immune-related adverse events: Review of the literature and suggested prophylactic strategy. J ImmunoTher. Cancer. (2020) 8:e000604. doi: 10.1136/jitc-2020-000604 32532839 PMC7295425

